# Characteristics and Outcomes of Paroxysmal Sympathetic Hyperactivity in Anti-NMDAR Encephalitis

**DOI:** 10.3389/fimmu.2022.858450

**Published:** 2022-04-06

**Authors:** Zhongyun Chen, Yan Zhang, Xiaowen Wu, Huijin Huang, Weibi Chen, Yingying Su

**Affiliations:** ^1^Department of Neurology, Xuanwu Hospital, Capital Medical University, Beijing, China; ^2^Department of Neurology, The Second Affiliated Hospital of Hainan Medical University, Haikou, China

**Keywords:** anti-N-methyl-D-aspartate receptor, encephalitis, autonomic dysfunction, paroxysmal sympathetic hyperactivity, outcome, therapeutic effect

## Abstract

**Background:**

To explore the clinical characteristics and prognosis of autonomic dysfunction and paroxysmal sympathetic hyperactivity (PSH), and evaluate the efficacy of drugs used to suppress PSH episode in anti-NMDAR encephalitis patients.

**Methods:**

Patients who met the diagnostic criteria of anti-NMDAR encephalitis were enrolled from January 2012 to August 2018 and followed up for 2 years. PSH was diagnosed according to the PSH-Assessment Measure. The demographics data, clinical features, auxiliary tests results, treatments, and outcomes were prospective collected and analyzed.

**Results:**

A total of 132 anti-NMDAR encephalitis patients were enrolled, of which 27.3% and 9.1% experienced autonomic dysfunction and probable PSH respectively. Cardiac autonomic dysfunction was the most common subtype (77.8%). Patients with a higher incidence of ovarian teratoma, mechanical ventilation, neurological intensive care unit admission, and elevated glucose and NMDAR antibody titer in the CSF were more likely to exhibit autonomic dysfunction or PSH. Episodes of PSH can be suppressed by monotherapy in patients without prior sedative drug use with an efficacy of 90%. No significant difference was observed between the prognosis of patients with or without autonomic dysfunction, or between the PSH versus non-PSH groups after 6 months and even during long-term follow-up. However, patients with cardiac autonomic dysfunction had poor prognosis at 6 months.

**Conclusion:**

PSH is a common clinical condition in patients with anti-NMDAR encephalitis, especially in severe cases, and can be effectively managed by several drug monotherapies. Despite necessitating longer hospital stay, autonomic dysfunction or PSH do not seem to compromise the neurological recovery of patients.

## Background

Anti-N-methyl-D-aspartate receptor (NMDAR) encephalitis is an autoimmune disorder characterized by the generation of autoantibodies against neuronal or synaptic antigens (1). Although early diagnosis and immunosuppressive therapy can improve the outcomes, 7.3-22% of the patients have poor prognosis during follow-up and the mortality rate ranges from 2.7-11.5% (2–5). Studies show that altered consciousness, intensive care unit (ICU) admission and lack of immunotherapy are associated with short-term poor prognosis (6).

The autonomic nervous system (ANS) controls all unconscious and involuntary functions in response to external stimuli in order to maintain homeostasis, and any disruption in one or more its branches affect daily life functions, and worsens the prognosis of several diseases. Severe dysfunction of the ANS may even lead to disability (7, 8). Approximately 10% to 50% of the anti-NMDAR encephalitis patients have autonomic dysfunction (2, 9), including tachycardia/bradycardia, hypertension/hypotension, gastrointestinal dysfunction, urinary dysfunction, abnormal pupil movement etc., which increase morbidity and mortality, complicate intensive care and can lead to hemodynamic shock (10). However, little is known regarding the correlation between autonomic instability and the prognosis of autoimmune encephalitis (4, 11). In addition, the prevalence and characteristics of autonomic dysfunction in anti-NMDAR encephalitis have not been systematically evaluated.

Paroxysmal sympathetic hyperactivity (PSH), also known as paroxysmal autonomic instability with dystonia, is characterized by hypertension, tachycardia, tachypnea, diaphoresis, agitation and dystonic posturing. It is caused by severe brain injury and associated with higher morbidity, longer hospital stays and worse outcomes (12, 13). PSH is often unrecognized in patients without traumatic brain injury (TBI), which has limited the development of specific management strategies. Only a few studies have reported an association between anti-NMDAR encephalitis and PSH (14). To this end, we analyzed the clinical and two-year outcomes of anti-NMDAR encephalitis patients at our center to identify the characteristics, predictors and long-term outcomes of autonomic dysfunction and PSH, and evaluate the efficacy of drugs used to suppress PSH episodes.

## Methods

### Patient Recruitment

This is a prospective analysis of Anti-NMDAR encephalitis patients recruited at the Department of Neurology of Xuanwu Hospital, Capital Medical University between January 2012 and August 2018 based on the following inclusion criteria (1): (1) age ≥14 years, (2) acute or subacute onset symptoms of encephalitis (less than three months), (3) exhibiting abnormal behavior or cognitive dysfunction, speech dysfunction, seizures, movement disorder, decreased level of consciousness, autonomic dysfunction or central hypoventilation, or a combination of the above symptoms (4) presence of IgG anti-GluN1 NMDAR antibodies in the cerebrospinal fluid (CSF) with or without serum positivity, and (5) absence of viral encephalitis, brain tumor, metabolic disease, drug poisoning etc. The exclusion criteria were as follows: (1) non-compliance with the treatment, (2) presence of other autoimmune or neurological paraneoplastic antibodies, and (3) not the first onset of anti-NMDAR encephalitis.

The patients were also stratified into the autonomic dysfunction and non-autonomic dysfunction groups, as well as the PSH and non-PSH groups. At the beginning of the study, a diagnosis of PSH was made according to the criteria proposed by Alejandro et al. (15) and subsequently confirmed using the PSH-Assessment Measure (PSH-AM) based on the Clinical Characteristics Scale (CFS) and the Diagnostic Likelihood Tool (DLT) proposed by Baguley et al. (16). The clinical features were classified as mild (1-6), moderate (7-12) and severe (>12) according to the CFS. Based on the sum of CFS and DLT scores, the likelihood of PSH was determined as unlikely (< 8), possible (8–16) and probable (≥17). Autonomic dysfunctions include cardiac autonomic dysfunction (e.g., tachycardia, bradycardia, malignant arrhythmia, hypertension, hypotension), gastrointestinal dysfunction (e.g., gastrointestinal motility insufficiency, constipation, gastropareses, nausea, and vomiting), hypersalivation, sudomotor dysfunction (e.g., anhidrosis, hyperhidrosis), fever, bladder dysfunction (e.g., urinary frequency, urgency and nocturia, urinary retention, urinary incontinence), and others (e.g., pupillary abnormalities, priapism). Further, the autonomic dysfunction group was further divided into the sympathetic (tachycardia, hypertension, bladder and gastrointestinal dysfunction), parasympathetic (bradycardia, generalized warmth, gastrointestinal hyperactivity and increased glandular secretion) and combined subgroups. Tachycardia and bradycardia were defined as heart rate >100 beats/min and <60 beats/min, hypertension and hypotension were defined as systolic blood pressure (SBP) >140 mm Hg or diastolic blood pressure (DBP) > 90 mm Hg and SBP< 90 mmHg or DBP< 60 mm Hg and fever was defined as temperature >37.5°C. The duration of these clinical signs needs to be at least 10 minutes and in the absence of other potential causes (e.g., underlying disease, medication, infection, pain). Patients who were admitted to the neurological intensive care unit (NCU) met at least one of the following criteria: respiratory failure requiring mechanical ventilation, impaired consciousness (GCS ≤ 12), or status epilepticus.

### Data Collection

The following demographic data and ancillary tests results were collected and analyzed: age of onset, sex, prodromal symptoms (including fever, headache, respiratory symptoms, emesis and diarrhea), clinical characteristics, time of admission, medical history, CSF analysis (e.g., pressure of lumbar puncture, white blood cell counts, and the levels of protein, glucose and chloride), brain magnetic resonance imaging (MRI) and electroencephalography (EEG) findings, treatment details and follow-up data. Serum and CSF antibodies were measured using indirect immunofluorescence test (IIFT) kits (EUROIMMUN AG, Lübeck, Germany) according to the manufacturer’s instructions. Samples were classified as strong positive (1:100 and above), positive (1:32), weak positive (1:10) and negative according to the antibody titers in serum and CSF. EEGs performed during the peak stage of the disease (14–60 days after the onset of symptoms) were analyzed for epileptic discharges, slow activity and other symptoms (including polymorphic delta rhythm and diffuse beta activities).

### Treatment

All patients were screened for tumors, symptomatic support and immunotherapy. Patients with cancer, such as ovarian teratoma, underwent surgical resection. Immunotherapies included intravenous glucocorticoid (1000 or 500mg methylprednisolone for 3 or 5 days followed by a gradual decrease in dosage), intravenous gamma immunoglobulin (IVIG; 0.4g/kg/day, 5 days per course), plasma exchange (PE; 3-5 times per course) or immunosuppressants (e.g., rituximab, cyclophosphamide, Moffett or azathioprine). For each probable PSH episode requiring intravenous pharmacological treatment, the drugs (e.g., Midazolam, Propofol, Dexmedetomidine, Diazepam, Phenobarbital) and their respective doses were selected based on the physician’s experience rather than objective evidence. Each drug administration was classified as fully effective or ineffective (or partially effective) based on whether the PSH episode was suppressed or not within 30 minutes.

### End Points

The patients were followed-up 6, 12 and 24 months after admission. Treatment efficacy and long-term outcomes were assessed using the modified Rankin Scale (mRS). Recurrence was defined as worsening of previous symptoms or the occurrence of new symptoms after two months of stabilization (5). Good and poor long-term outcomes were respectively defined as mRS scores 0-2 and 3-6.

### Statistical Analysis

Statistical analyses were performed using SPSS 20.0 (IBM Corporation, Armonk, NY, USA). Quantitative data with normal distributions are presented as mean ± SD, whereas data with non-normal distributions are presented as median with the interquartile range (IQR). Student’s t test was used to compare data with normal distribution and homogeneous variance, and Mann–Whitney U test was used to evaluate differences in ranked data. Categorical data were summarized as counts (percentages), and compared by Pearson chi-square test or Fisher exact test. Multiple imputation by chained equations was used to address missing data. P values < 0.05 were considered statistically significant.

## Results

### Patient Characteristics

**Figure 1:**
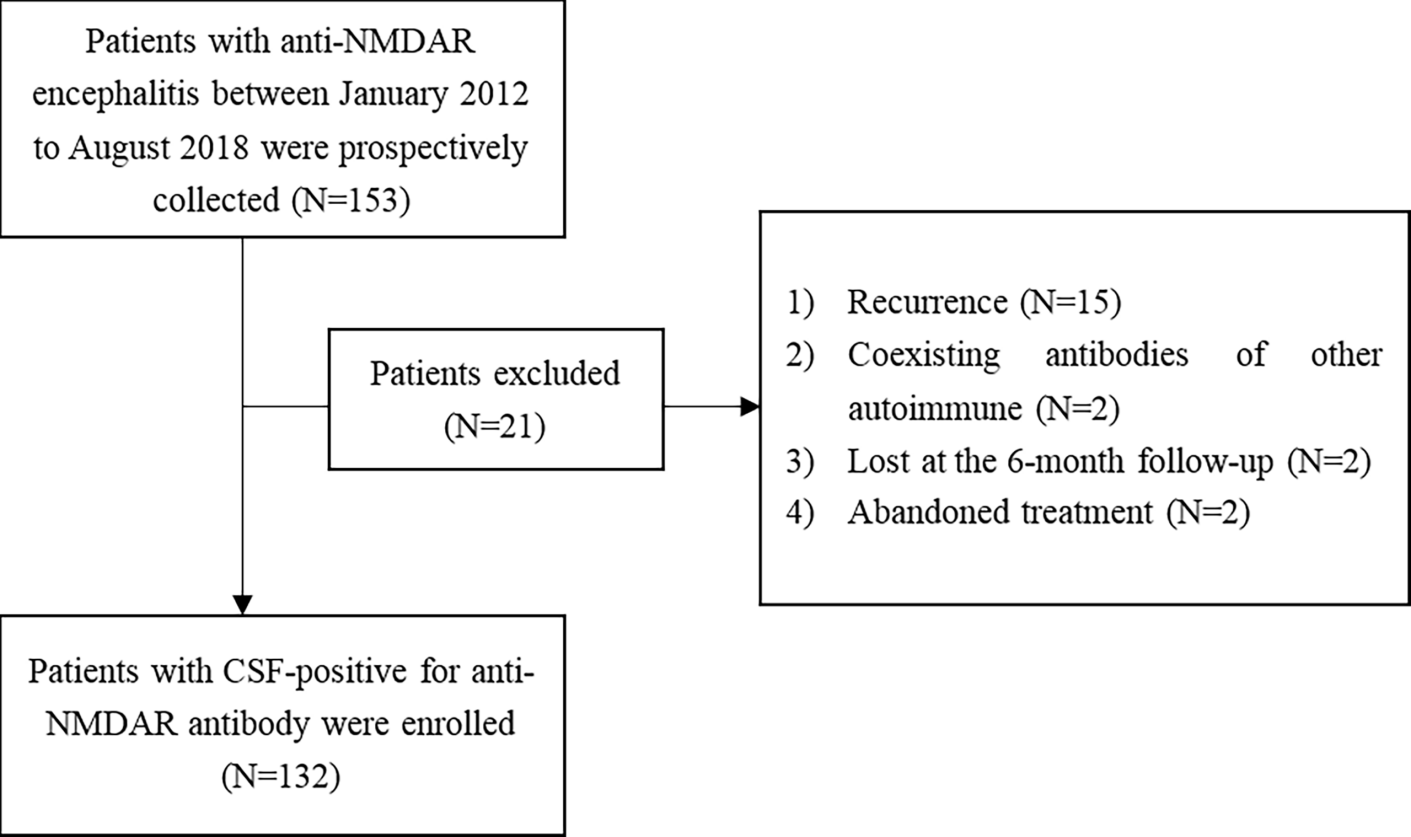
Patient inclusion/exclusion flowchart.

A total of 153 patients met the inclusion and exclusion criteria of our study (**Figure 1**).

The median age at onset was 25 (IQR 19–34) years, and 59 (44.7%) patients were females.

### The Characteristics of Autonomic Dysfunction

**Figure 2:**
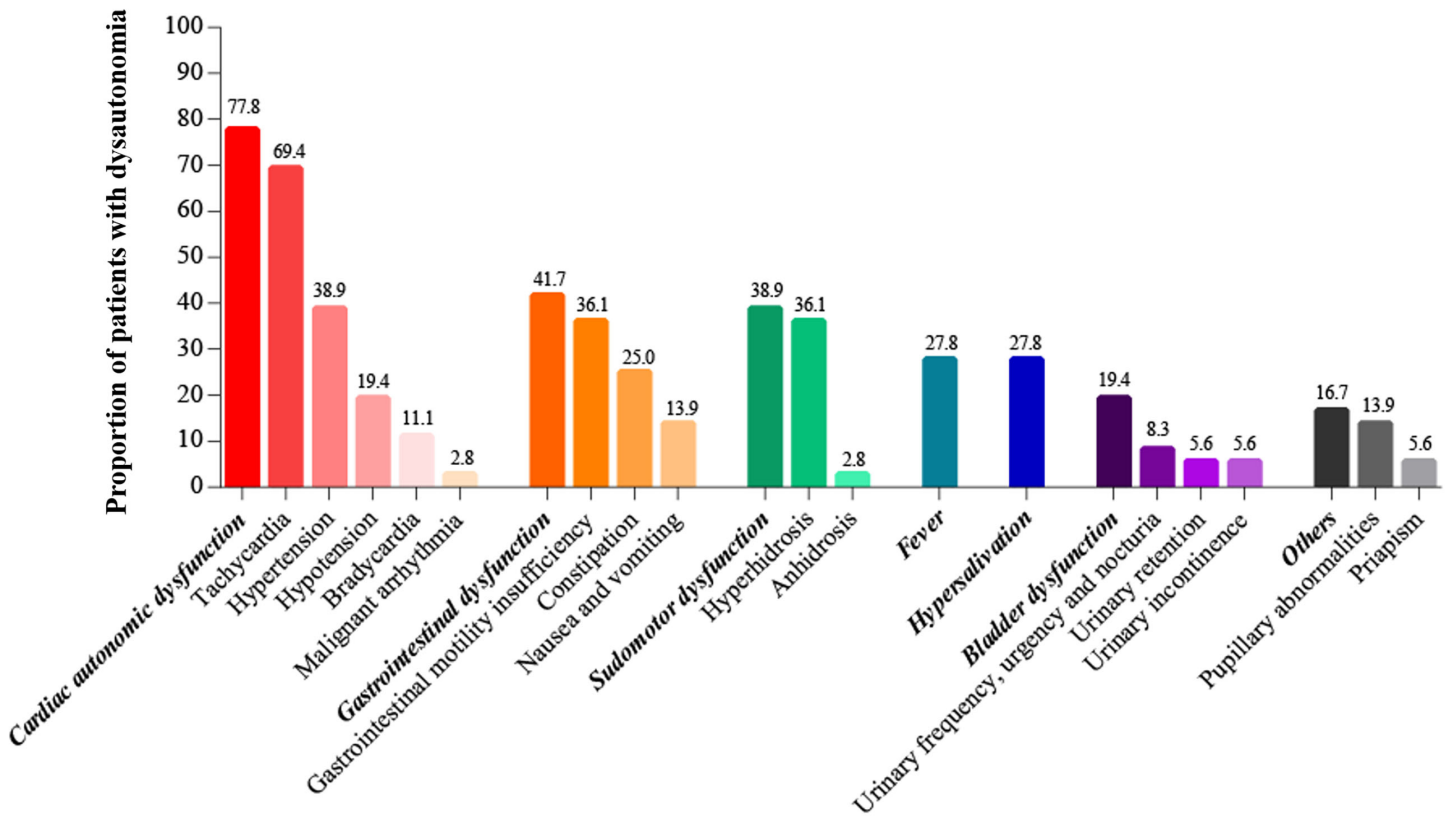
The incidence of autonomic dysfunction subdivisions.

The overall incidence of autonomic dysfunction was 27.3% (36/132), with more than half of the patients (52.8%, 19/36) exhibiting both sympathetic and parasympathetic dysfunction. The incidence of pure sympathetic and parasympathetic dysfunction were 25% and 22.2% respectively. Cardiac autonomic dysfunction, gastrointestinal dysfunction and sudomotor dysfunction were most common, with respective incidence rates of 77.8%, 41.7% and 38.9% (**Figure 2**).

### The Characteristics of PSH

According to the PSH-AM criteria, 12 (9.7%) patients were diagnosed with probable PSH, of which 11 were admitted to the NCU. As shown in **Table 1**, the most common symptoms of PSH were tachycardia (100%), tachypnea (100%) and posturing (100%), followed by hyperhidrosis (75%), hyperthermia (50%) and hypertension (42%). The median maximum CFS and DLT scores were 12 (10, 13.5) and 8 (7, 8.8) respectively. According to the CFS scores, 8 (67%) patients had moderate PSH and 4 patients exhibited severe PSH. The median (IQR) time from onset to PSH and the duration of PSH were 25.5 (16.0, 46.8) and 17.0 (7.3, 24.0) days respectively. Eight patients were co-infected (pulmonary infection) and 9 patients were receiving immunomodulators.

**Table 1:** Clinical features of patients with PSH (n = 12).

Case	Sex	The onset age	Tachycardia	Tachypnea	Hypertension	Hyperthermia	Hyperhidrosis	Dystonia	CFS scores	Time from onset to PSH (days)	Infection	Diabetes mellitus	Immunotherapy	Time from initiating immunotherapy to PSH	PSH duration (days)
1	F	27	+	+	+	+	–	+	12	13	–	–	+	Day 3 of steroid and IVIG therapy	8
2	F	21	+	+	+	–	–	+	10	20	+	–	+	Day 6 of steroid and day 7 of IVIG therapy	24
3	M	31	+	+	+	+	+	+	14	46	+	–	+	Day 16 of steroid and IVIG therapy	5
4	F	25	+	+	–	+	+	+	12	62	–	–	+	Day 27 of steroid and IVIG therapy	22
5	F	16	+	+	–	+	+	+	12	15	–	–	–	–	15
6	M	16	+	+	–	–	+	+	12	47	+	–	+	Day 12 of steroid and day 7 of IVIG therapy	24
7	F	19	+	+	+	–	+	+	14	34	+	–	+	Day 2 of steroid therapy	35
8	F	31	+	+	–	–	+	+	9	56	–	–	+	Day 15 of steroid and day 37 of IVIG therapy	17
9	F	23	+	+	+	+	+	+	15	16	+	–	+	Day 3 of IVIG	123
10	M	14	+	+	–	+	–	+	10	25	+	–	+	Day 5 of steroid therapy and IVIG.	17
11	F	32	+	+	–	–	+	+	11	16	+	–	–	–	7
12	M	23	+	+	–	–	+	+	10	26	+	–	–	–	4

CFS, Clinical Feature Scale; IVIG, intravenous immunogloblin; PSH, paroxysmal sympathetic hyperactivity.

### Comparison of Clinical and Ancillary Features Between Patients With or Without Autonomic Dysfunction or PSH

As shown in **Table 2**, patients with autonomic dysfunction or PSH had a higher incidence of ovarian teratoma, involuntary movement, impaired consciousness, central hypoventilation, mechanical ventilation and NCU admission compared to patients without ANS involvement. Autonomic dysfunction/PSH was also associated with elevated glucose levels and NMDAR antibody titers in the CSF, as well as a higher rate of IVIG, plasma exchange and immunosuppressor treatments (P<0.05).

**Table 2:** Demographics, clinical manifestations, auxiliary test results of patients with anti-NMDAR encephalitis.

	Total (n=132)	Non-autonomic dysfunction group (n=96)	Autonomic dysfunction group (n=36)	P values	Non-PSH group (n=120)	PSH group (n=12)	P values
Age, years, median (IQR)	25.0 (19.0,34.0)	25.5 (19.0,36.0)	23.0 (17.3,30.5)	0.098	25.0 (19.0,35.8)	23.0 (16.8,30.0)	0.199
Female, n (%)	59 (44.7)	41 (42.7)	18 (50.0)	0.453	51 (42.5)	8 (66.7)	0.193
Ovarian teratoma, n (%)	9 (6.8)	3 (3.1)	6 (16.7)	0.018	5 (4.2)	4 (33.3)	0.001
Prodromal symptoms, n (%)	69 (52.3)	44 (45.8)	25 (69.4)	0.016	63 (52.5)	6 (50.0)	0.869
Clinical manifestations, n (%)							
Mental behavior disorder	98 (74.2)	66 (68.8)	32 (88.9)	0.018	87 (72.5)	11 (91.7)	0.271
Epileptic seizure	88 (66.7)	61 (63.5)	27 (75.0)	0.214	77 (64.2)	11 (91.7)	0.108
Involuntary movement	62 (47.0)	35 (36.5)	27 (75.0)	0.000	50 (41.7)	12 (100)	0.000
Language impairment	33 (25.0)	25 (26.0)	8 (22.2)	0.652	30 (25.0)	3 (25.0)	1.000
Impaired consciousness	63 (47.7)	34 (54.0)	29 (80.6)	0.000	52 (43.3)	11 (91.7)	0.001
Cognitive impairment	42 (31.8)	31 (32.3)	11 (30.6)	0.849	38 (31.7)	4 (33.3)	1.000
Central hypoventilation	30 (22.7)	5 (5.2)	25 (16.7)	0.000	24 (20.0)	6 (50.0)	0.045
Electroencephalogram, n=105, n (%)							
Normal	10 (9.5)	8 (10.7)	2 (6.7)	0.528	11 (11.7)	1 (9.1)	1.000
Epileptic discharges	24 (22.9)	14 (18.7)	10 (33.3)	0.106	20 (21.3)	4 (36.4)	0.454
Slow activity	57 (54.3)	43 (57.3)	14 (46.7)	0.322	53 (56.4)	4 (36.4)	0.207
others	14 (13.3)	10 (13.3)	4 (13.3)	1.000	10 (9.6)	4 (36.4)	0.036
Cranial MRI, n (%)							
Normal	51 (38.6)	33 (34.4)	18 (50.0)	0.101	45 (37.5)	6 (50.0)	0.591
Temporal lobe	33 (25.0)	21 (21.9)	12 (33.3)	0.176	30 (25.0)	3 (25.0)	1.000
Limbic lobe	47 (35.6)	33 (34.4)	14 (38.9)	0.630	42 (35.0)	5 (41.7)	0.886
Frontal lobe	30 (22.7)	21 (21.9)	9 (25.0)	0.703	26 (21.7)	4 (33.3)	0.577
Parietal lobe	15 (11.4)	10 (10.4)	5 (13.9)	0.576	13 (10.8)	2 (16.7)	0.896
Occipital lobe	12 (9.1)	8 (8.3)	4 (11.1)	0.621	11 (9.2)	1 (8.3)	1.000
Insular lobe	18 (13.6)	10 (10.4)	8 (22.2)	0.078	15 (12.6)	3 (25.0)	0.454
Diencephalon	4 (3.0)	3 (3.1)	1 (2.8)	1.000	4 (3.3)	0 (0)	1.000
Cerebellum	3 (2.3)	3 (3.1)	0 (0)	0.562	3 (2.5)	0 (0)	1.000
Brainstem	9 (6.8)	6 (6.3)	3 (8.3)	0.672	9 (7.5)	0 (0)	0.702
CSF analysis							
Opening pressure, mmH_2_O, median (IQR)	180.0 (140.0,225.0)	180.0 (135.0,220.0)	200.0 (147.5,240.0)	0.151	180.0 (140.0,220.0)	180.0 (142.5,260.0)	0.672
WBC, ×10^6^/L, median (IQR)	15.0 (5.0,32.0)	13.5 (5.0,28.0)	23.0 (10.0,37.0)	0.056	14.0 (5.0,30.0)	29.0 (14.5,35.8)	0.161
Protein, mg/dl, median (IQR)	31.0 (20.3,44.0)	31.0 (20.5,44.0)	31.0 (20.0,44.0)	0.923	31.0 (21.0,44.0)	26.0 (10.5,41.5	0.218
Glucose, mg/dl, median (IQR)	60.8 (54.5,69.4)	59.4 (54.1,66.4)	68.7 (56.9,79.8)	0.004	60.0 (54.5,68.2)	70.6 (64.9,79.2)	0.005
Chloride, mmol/L, median (IQR)	121.0 (117.0,124.0)	121.0 (117.0,124.0)	121.0 (117.0,125.0)	0.984	121.0 (117.0,124.0)	120.0 (117.3,124.5)	0.662
Serum, Glucose, mmol/L, median (IQR)	5.0 (4.5,5.7)	5.0 (4.5,5.4)	5.4 (4.5,6.1)	0.180	4.5 (4.5,5.6)	5.3 (4.5,5.9)	0.159
CSF NMDAR antibody titers, n (%)							
+	15 (11.4)	13 (13.5)	2 (5.6)	0.198	15 (12.5)	0 (0)	0.410
++	74 (56.1)	59 (61.5)	15 (41.7)	0.041	70 (58.3)	4 (33.3)	0.096
+++	43 (32.6)	24 (25.0)	19 (52.8)	0.002	35 (29.2)	8 (66.7)	0.020
Serum NMDR antibody titers, n (%)							
-	71 (53.8)	56 (58.3)	15 (41.7)	0.087	68 (56.7)	3 (25.0)	0.036
+	21 (15.9)	14 (14.6)	7 (19.4)	0.496	18 (15.0)	3 (25.0)	0.625
++	34 (25.8)	24 (25.0)	10 (27.8)	0.745	29 (24.2)	5 (41.7)	0.329
+++	6 (4.5)	2 (2.1)	4 (11.1)	0.047	5 (4.2)	1 (8.3)	1.000
Mechanical ventilation, n (%)	29 (22.0)	12 (12.5)	17 (47.2)	0.000	21 (17.5)	8 (66.7)	0.000
ICU admission, n (%)	43 (32.6)	19 (19.8)	24 (66.7)	0.000	32 (26.7)	11 (91.7)	0.000

CSF, cerebrospinal fluid; ICU, intensive care unit; IQR, interquartile range; mRS, modified Rankin Scale; MRI, magnetic resonance imaging; NMDAR, N-methyl-D-aspartate receptor; PSH, paroxysmal sympathetic hyperactivity; WBC, white blood cell.

### Efficacy of Drugs Against PSH Episodes

There were 57 probable PSH episodes requiring intravenous pharmacological treatment, of which 18 (31.6%) were related to stimuli, such as turning, back-patting and suctioning. Diazepam and phenobarbitone were commonly administered to control PSH in patients without previous sedative use, and the overall efficacy was 90%. However, the efficacy of monotherapy dropped to 69.6% in patients with previous sedative use, and approximately half of the episodes needed a combination of drugs to control the symptoms (**Table 3**).

**Table 3:** Efficacy of drugs used to suppress PSH episodes.

Drug treatment	Dose range	Efficacy, %
**Without Prior Sedative drugs**		
**Monotherapy**		
Overall		9/10 (90.0)
Diazepam	5-10 mg	7/8 (87.5)
Phenobarbitone	0.2 mg	2/2 (100)
**With Prior Sedative drugs**		
**Monotherapy**		
Overall		16/23 (69.6)
Propofol	30-60 mg/h	4/6 (66.7)
Phenobarbitone	0.2 mg	4/5 (80.0)
Midazolam	2-6 mg/h	4/6 (66.7)
Diazepam	10 mg	3/4 (75.0)
Dexmedetomidine	16-20ug/h	1/2 (50)
**Combination drug therapy**		
Overall		17/24 (70.8)
Midazolam + Phenobarbitone	3-6mg/h + 0.2mg	4/6 (66.7)
Propofol + Phenobarbitone	30-60mg/h + 0.2mg	4/5 (80.0)
Dexmedetomidine+ Phenobarbitone	16-20ug/h + 0.2mg	3/4 (75.0)
Propofol + Midazolam	40-60mg/h +2-4mg/h	3/4 (75.0)
Midazolam + Dexmedetomidine	2-5mg/h + 16-20ug/h	2/3 (66.7)
Dexmedetomidine+ Propofol	12-20ug/h +40-60mg/h	1/2 (50.0)

PSH, paroxysmal sympathetic hyperactivity.

### Prognosis of Autonomic Dysfunction and PSH

**Figure 3:**
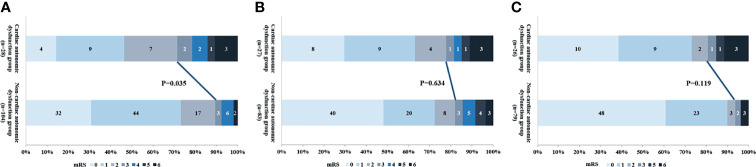
The relationship between cardiac autonomic dysfunction and outcome at **(A)** 6 months, **(B)** 12 months and **(C)** 24 months.

The median duration of hospital stay was longer in patients with autonomic dysfunction or PSH compared to the respective control groups [median (IQR): 38.5(21.3 to 72.3) days vs. 16.0 (12.0 to 21.0) days, P<0.000; 63.5(36.8 to 81.5) days vs.18.0(13.0 to 27.9) days, P<0.000]. There was no significant difference between the recurrence and functional outcomes at 6, 12 and 24 months across all groups (**Table 4**). In addition, cardiac autonomic dysfunction was associated with poor prognosis at 6 months (28.6% vs. 9.0%, P=0.035; 19.2% vs. 6.3%, P=0.019; **Figure 3**), whereas the other subtypes of autonomic dysfunction were not associated with patient prognosis.

**Table 4:** Therapy and prognosis of patients with anti-NMDAR encephalitis.

	Total (n=132)	Non-autonomic dysfunction group (n=96)	Autonomic dysfunction group (n=36)	P values	Non-PSH group (n=120)	PSH group (n=12)	P values
Immunotherapy, n (%)							
Steroids	113 (85.6)	79 (82.3)	34 (94.4)	0.076	102 (85.0)	11 (91.7)	0.845
IVIG	77 (58.3)	45 (46.9)	32 (88.9)	0.000	65 (54.2)	12 (100)	0.002
Plasma exchange	23 (17.4)	8 (8.3)	15 (41.7)	0.000	16 (13.3)	7 (58.3)	0.000
Immunosuppressor	26 (19.7)	13 (13.5)	13 (36.1)	0.004	21 (17.5)	5 (41.7)	0.104
Outcome							
Length of ICU length of stay, n=43, days, median (IQR)	38 (18.0,73.0)	23.0 (13.0,54.0)	39.0 (25.8,78.3)	0.106	29.0 (16.5,55.0)	61.0 (36.0,82.0)	0.079
Hospital length of stay, days, median (IQR)	18.0 (13.0,33.5)	16.0 (12.0,21.0)	38.5 (21.3,72.3)	0.000	18.0 (13.0,27.9)	63.5 (36.8,81.5)	0.000
Hospital mortality, n (%)	5 (3.8)	2 (2.1)	3 (8.3)	0.125	5 (4.2)	0 (0)	1.000
Recurrence, n (%)	14 (10.6)	11 (11.5)	3 (8.3)	0.604	13 (10.8)	1 (8.3)	1.000
mRS>2 after 6 months, n (%)	19 (14.4)	11 (11.5)	8 (22.2)	0.117	17 (14.2)	2 (16.7)	1.000
mRS>2 after 12 months, n=110, n (%)	21 (19.1)	14 (18.2)	7 (21.2)	0.711	20 (20.2)	1 (9.1)	0.628
mRS>2 after 24 months, n=105, n (%)	10 (9.5)	5 (6.8)	5 (15.6)	0.159	9 (8.5)	11 (10.0)	1.000

ICU, intensive care unit; IQR, interquartile range; IVIG, IV immunoglobulin; mRS, modified Rankin Scale; NMDAR, N-methyl-D-aspartate receptor; PSH, paroxysmal sympathetic hyperactivity.

## Discussion

The overall prevalence of autonomic dysfunction and PSH in our cohort of anti-NMDAR encephalitis patients were 27.3% and 9.1% respectively, and were associated with a higher incidence of ICU admission, mechanical ventilation, ovarian teratomas, along with elevated NMDAR antibody titers and glucose levels in the CSF. Most PSH episodes were suppressed by acute drug administration, and longer hospital stays did not compromise the neurological recovery of these patients. However, cardiac autonomic dysfunction was associated with poor outcomes at 6 months.

PSH in particular is the main clinical manifestation of severe TBI, hypoxic brain injury and stroke, orthostatic hypotension, thermoregulatory disorders and detrusor hyperactivity (17). The prevalence of PSH in patients with TBI ranges from 7.7 to 32.6% among various cohorts (18, 19). In addition, Raquel et al. reported PSH in 40.7% of the patients with meningoencephalitis and/or encephalitis in the pediatric ICU (20). In the present study, the overall prevalence of PSH was 9.1%, which increased to 25.6% in the patients admitted to the NCU. Infections, seizures and drug fever need to be excluded before confirming the diagnosis of PSH. PSH and infections include similar symptoms such as tachycardia, tachypnea, and fever, making it difficult to distinguish between the two. In general, though, there are some minor variances. To begin with, the duration varies, with infection-related symptoms often persistent, but PSH-related symptoms are episodic and easily triggered by external stimuli (21). Second, the accompanying symptoms vary, with PSH usually accompanied by posturing and pupillary dilation (22). Third, therapeutic response varies; antibiotics cannot relieve symptoms caused by PSH, necessitating the use of PSH-relieving medications (23). A case series has demonstrated the usefulness of measuring serum procalcitonin levels in distinguishing PSH from infectious etiology and managing two distinct clinical entities (24). Four PSH patients in this study had epileptic discharges during the disease, similar to the incidence of non-PSH patients. While no epileptic discharges were found in 9 patients with PSH episodes who undergoing EEG monitoring. Drug reactions, such as malignant hyperthermia or neuroleptic malignant syndrome, are mostly associated with specific drugs like dopamine receptor blockers or nondepolarizing muscle relaxants, which were not used in our study. In addition, PSH episodes tend to be confused with or superimposed on central hypoventilation or involuntary movements in anti-NMDAR encephalitis. Given the high sensitivity and low specificity of the diagnostic criteria for PSH-AM (25), the true incidence of PSH may be overestimated. However, the strict inclusion criteria of this study, in which all patients met the probable PSH, reduced the possibility of misdiagnosis. The most common symptoms of PSH in our cohort were tachycardia (100%), tachypnea (100%) and posturing (100%), which contradicts previous reports indicating that PSH after TBI and ICH mainly manifest as hypertonia (94%) and hyperhidrosis (77%) (21), or hyperthermia (80%) and hyperhidrosis (80%) (26).

Autonomic dysfunction and PSH can result from the impairment in the central autonomic regulatory centers, such as insular cortex, anterior cingulate and ventral prefrontal regions, as well as lower centers located in the amygdala, hypothalamus, thalamus, brainstem and spinal cord (12, 27, 28). Although structural lesions that increase the likelihood of autonomic instability or PSH have been identified (29), we did not detect any association between structural anomalies and PSH. However, patients with autonomic dysfunction showed a higher propensity for insular lobe abnormalities, suggesting involvement of autonomic centers at the molecular level. In addition, there is evidence that PSH is caused by the disruption of multiple sympathetic circuits rather than a single lesion (27, 30). Common risk factors of autonomic dysfunction/PSH are younger (31) or older age (13), tracheostomy (32), lower GCS scores on admission (29), higher grade of diffuse axonal injury (19, 33) and deep parenchymal lesions (29). In this study, ICU admission, mechanical ventilation, ovarian teratoma (34) and elevated CSF NMDAR antibody titers (31) were all strongly associated with disease severity, and more prevalent in patients with autonomic dysfunction and PSH. Interestingly, the levels of CSF glucose were significantly higher in patients with autonomic instability or PSH, although the exact association remains unclear.

The PSH symptoms in almost 72% of the patients are caused by unavoidable non-noxious stimuli, such as turning, back-patting, suctioning and emotional excitement (35). In our study, these stimuli were the cause of 31.6% of the PSH cases. Currently, intravenous anesthetics, β-adrenergic blockers, α2-agonists and benzodiazepines are used to treat patients with PSH (12). However, the efficacy of these drugs has not been compared extensively. A retrospective cohort study on 26 PSH patients showed that the most commonly used analgesic drugs were not very effective whereas benzodiazepine drugs had satisfactory effects (36). In this study, we found that diazepam was frequently administered and highly effective in PSH patients without prior exposure to sedative drugs. However, the efficacy of monotherapy was lower in patients with a history of sedative drug use for controlling dysphoria, epilepsy or other conditions, and half of them needed a combination of drugs. This can be attributed to the development of drug tolerance in those patients. The combination of phenobarbitone with one anesthetic (dexmedetomidine, midazolam or propofol) is at present most common with relatively high efficacy.

Some studies show that autonomic instability or PSH is associated with worse functional outcomes of other diseases compared to patients without autonomic instability or PSH symptoms (20, 37–41). In this study, we found that patents with autonomic dysfunction or PSH had a longer ICU stay, as reported previously (31), although this did not appear to compromise their neurological recovery. While active treatment improved the prognosis of most patients in our cohort, cardiac autonomic dysfunction was associated with short-term poor prognosis, likely due to the greater susceptibility to hemodynamic instability in this group of patients. Consistent with our findings, Byun et al. showed that cardiac autonomic function, specifically sympathetic activity, was reduced and cardiac autonomic dysfunction was associated with poor function at 3 months in 11 patients with anti-NMDAR encephalitis (10).

Our study has some limitations that should be acknowledged. First, asymptomatic autonomic symptoms such as tachycardia, hypertension etc. can easily overlooked without electrocardiograph monitoring. Furthermore, some parasympathetic excitatory symptoms like hypohidrosis, xerostomia and xerophthalmia can be easily missed. Second, autonomic dysfunction is objectively measured using heart rate variability and pupillary dynamics (42), whereas our assessment was based on clinical presentation. Third, the drug treatments were typically chosen based on the physician’s experience rather than objective evidence, and the small sample size and relatively high number of drug subgroups prevented the comparison of the efficacy of different drugs. Further randomized clinical trials are needed to address the above points.

## Conclusions

PSH is a common clinical symptom in patients with anti-NMDAR encephalitis, especially in severe cases, and can be suppressed by active treatment. Combined immunotherapy can significantly improve patient prognosis despite longer hospital stays and challenging treatment.

## Data Availability Statement

The original contributions presented in the study are included in the article/supplementary material. Further inquiries can be directed to the corresponding author.

## Ethics Statement

The studies involving human participants were reviewed and approved by Ethics Committee of the Xuanwu Hospital, Capital Medical University. The patients/participants provided their written informed consent to participate in this study.

## Author Contributions

ZC enrolled the patients, performed statistical analysis and drafted the manuscript. XW and HH were involved in patient enrollment and data verification. WC performed data verification of data and statistical analysis. YZ and YS designed the study and helped draft the manuscript. All authors read and approved the final manuscript.

## Funding

This project was supported by the National Key Research and Development Program of China Research (2020YFC2005403) and by the Beijing Municipal Administration of Hospitals Incubating Program (PX2020035).

## Conflict of Interest

The authors declare that the research was conducted in the absence of any commercial or financial relationships that could be construed as a potential conflict of interest.

## Publisher’s Note

All claims expressed in this article are solely those of the authors and do not necessarily represent those of their affiliated organizations, or those of the publisher, the editors and the reviewers. Any product that may be evaluated in this article, or claim that may be made by its manufacturer, is not guaranteed or endorsed by the publisher.

## Abbreviations

ANS, autonomic nervous system; CSF, cerebrospinal fluid; ICU, intensive care unit; IQR, interquartile range; IVIG, IV immunoglobulin; mRS, modified Rankin Scale; NMDAR, N-methyl-D-aspartate receptor; PSH, paroxysmal sympathetic hyperactivity.
